# Exocrine Pancreatic Insufficiency Treated With Pancreatic Replacement Enzyme in a Premature Neonate: A Case Report

**DOI:** 10.7759/cureus.101045

**Published:** 2026-01-07

**Authors:** Benjamin A Hopkins, Pratishtha Chhabra

**Affiliations:** 1 Pediatrics, University of California San Francisco, Fresno, Fresno, USA

**Keywords:** exocrine pancreatic insufficiency, immobilized lipase, medium-chain triglycerides, neonate, pancreatic enzyme replacement therapy, pancreatic insufficiency, pancrelipase, premature, preterm

## Abstract

Exocrine pancreatic insufficiency (EPI) in neonates is rare and challenging to diagnose, particularly in extremely preterm infants in whom physiologic pancreatic immaturity may mimic disease. Persistent malabsorption, poor growth, and repeatedly abnormal fecal elastase-1 (FE1) levels can help differentiate true EPI from transient developmental insufficiency.

An extremely preterm female born at 25.0 weeks adjusted gestational age (AGA), weighing 775 g, developed feeding intolerance, direct hyperbilirubinemia, and growth failure during her neonatal intensive care unit course. Serial FE1 testing on days of life 47 and 67 showed persistently low values consistent with EPI. Despite nutritional optimization, including the use of medium-chain triglyceride (MCT)-containing formula and immobilized lipase, the patient exhibited inadequate weight gain. Pancreatic enzyme replacement therapy (PERT) with pancrelipase was initiated at 35.5 weeks AGA, leading to significant improvement in weight gain and normalization of direct bilirubin. Genetic testing identified three CFTR variants of uncertain clinical significance; parental studies confirmed carrier status, and rapid exome sequencing did not identify alternative etiologies. FE1 normalized by 40.6 weeks AGA, permitting discontinuation of PERT. The infant was medically stable for discharge at 42.5 weeks AGA following gastrostomy tube placement for feeding support.

This case underscores the diagnostic complexity of distinguishing EPI from physiologic enzyme immaturity in extremely preterm infants. FE1 served as a useful biomarker when interpreted in the context of gestational age and feeding status. Nutritional strategies, including MCT supplementation, PERT, and immobilized lipase, support growth; however, their use should be reserved for infants with objective evidence of EPI. The presence of CFTR variants of uncertain significance underscores the importance of cautious interpretation of genetic results and ongoing monitoring for the development of pancreatic dysfunction.

EPI should be considered in extremely preterm infants with persistent malabsorption and growth faltering beyond expected physiologic immaturity. Early identification through serial FE1 testing and targeted therapy can optimize nutritional outcomes. Further research is needed to clarify the clinical relevance of CFTR variants of uncertain significance and to refine evidence-based approaches to enzyme supplementation in this population.

## Introduction

Exocrine pancreatic insufficiency (EPI) is a deficiency of exocrine pancreatic enzymes or bicarbonate secretion in the neonatal period, resulting in impaired digestion and absorption of fats, proteins, and carbohydrates [1,2]. EPI can lead to clinical manifestations such as steatorrhea, failure to thrive, and malnutrition in affected neonates [1,2]. In neonates, EPI may be due to congenital disorders such as cystic fibrosis (CF), Shwachman-Diamond syndrome, Johanson-Blizzard syndrome, pancreatic agenesis, or pancreatic hypoplasia [3]. It is essential to distinguish this from the physiologic immaturity of pancreatic enzyme secretion seen in preterm infants. Physiologic pancreatic immaturity may cause transient fat malabsorption but does not meet the criteria for actual EPI unless it results in clinically significant malabsorption and failure to thrive [4,5]. We present a case of a 25-week adjusted gestational age (AGA) female diagnosed with EPI due to failure to thrive and confirmed after repeated abnormal levels of fecal elastase (FE1) on days of life 47 and 67.

## Case presentation

An extremely preterm, 25.0-week-old female weighing 775 grams (53rd percentile) was born by C-section, due to premature labor and intrauterine fetal intolerance, to a G3P0 woman who had received prenatal care. Pregnancy was complicated by breech presentation, preterm onset of labor, type 2 diabetes mellitus, obesity, asthma, cerclage failure, and a previous history of a 21-week fetal demise. The mother received one dose of antenatal steroids and one dose of antibiotics just before delivery. The mother had an unknown Group B *Streptococcus* (GBS) status, was Rh positive, antibody negative, and had otherwise negative serologic screening results. The newborn (NB) received positive-pressure ventilation (PPV) and was intubated at delivery due to respiratory failure secondary to a small pneumothorax. On admission, she had pulmonary insufficiency for which she was given surfactant and placed on a conventional ventilator. Umbilical venous catheters (UVCs) and umbilical arterial catheters (UACs) were placed to gain central line access. Labs at birth revealed an elevated lactic acid and an unremarkable complete blood count (CBC). Blood and respiratory cultures were also collected. The NB was started on ampicillin and gentamicin while blood cultures were pending, and received dextrose 10% parenteral nutrition (PN) until a customized total parenteral nutrition (TPN) solution was prepared.

**Figure 1:**
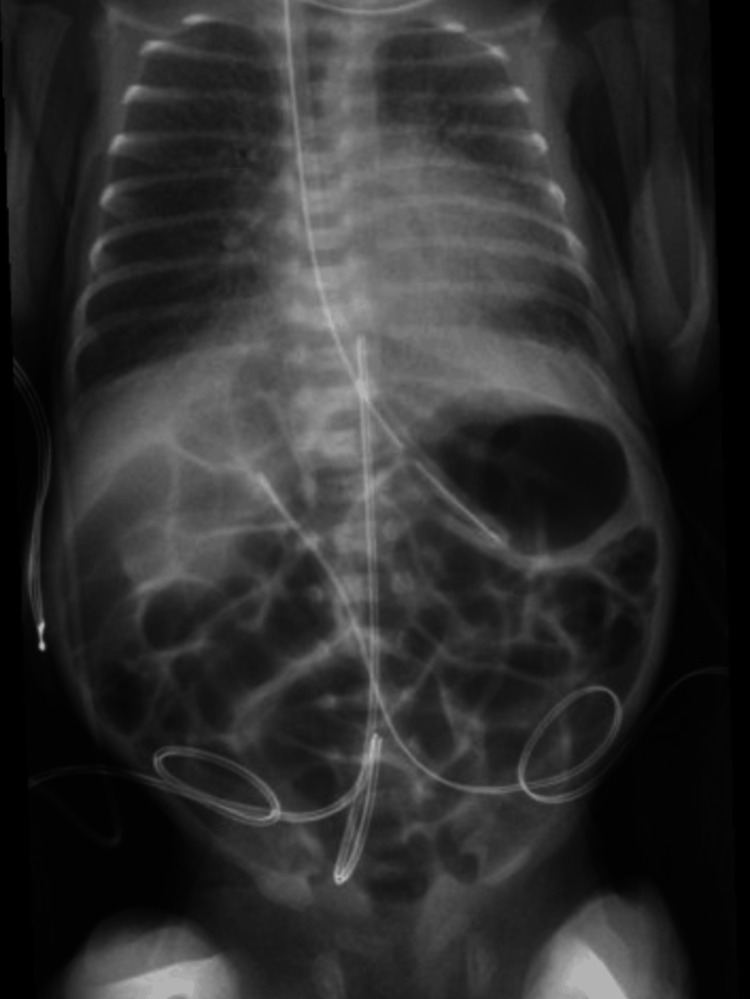
KUB radiograph showing gaseous distension of the stomach and small intestine without evidence of free intraperitoneal air or pneumatosis in the small intestinal wall KUB: kidney, ureter, and bladder

**Figure 2:**
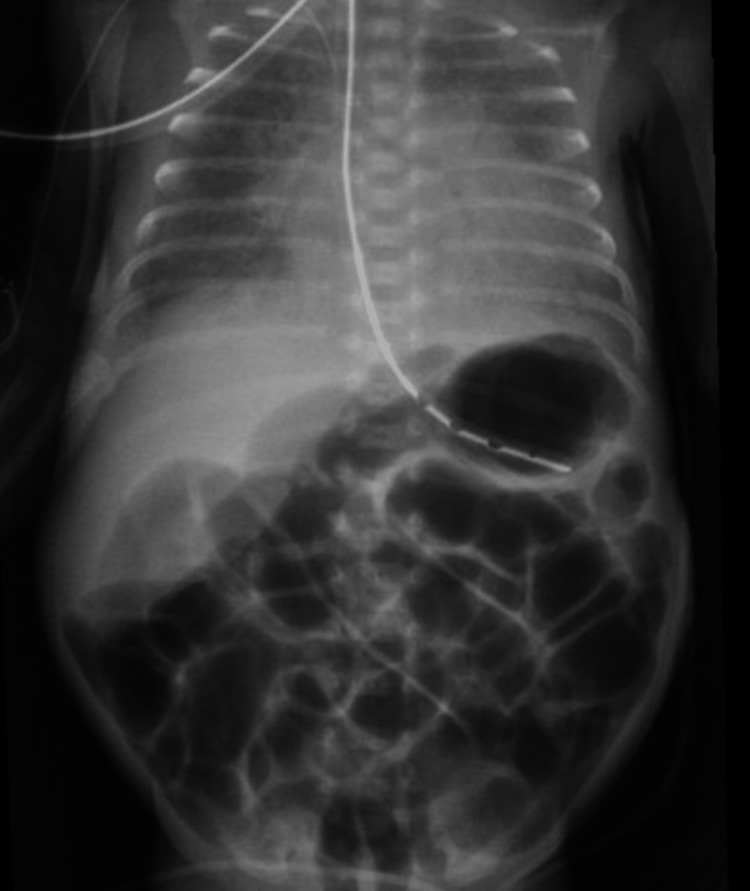
KUB radiograph showing worsening gaseous distension of the stomach and small intestine, without evidence of free intraperitoneal air or pneumatosis in the small intestinal wall KUB: kidney, ureter, and bladder

**Figure 3:**
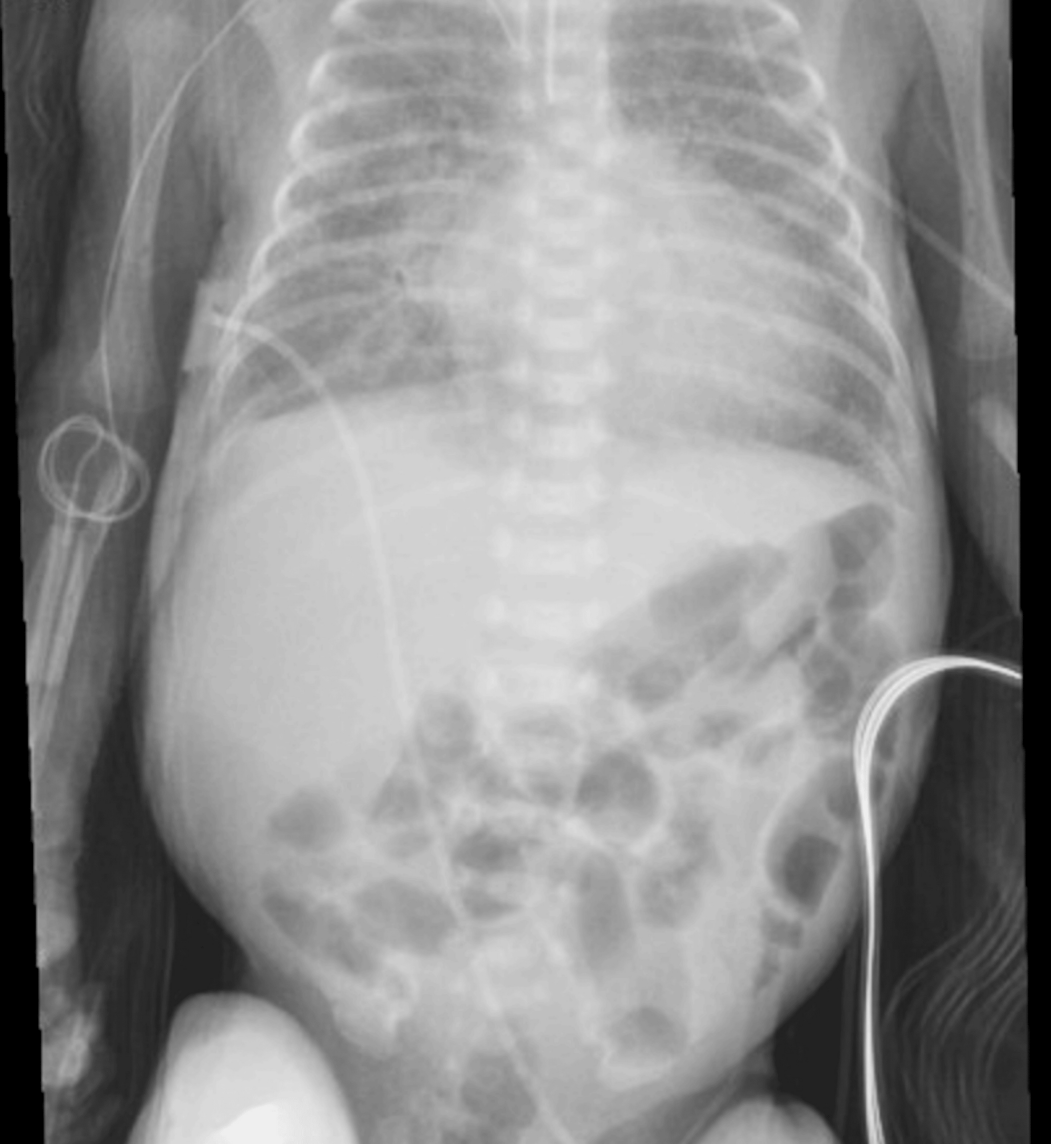
KUB radiograph showing improvement in gaseous distension of the stomach and small intestine following gastric tube decompression KUB: kidney, ureter, and bladder

At 25.5 weeks AGA, lipids were discontinued due to significant hypertriglyceridemia (Table 1). Later that day, feedings were stopped due to repeated emesis and feeding intolerance, and a kidney, ureter, and bladder (KUB) X-ray (Figure 1) was taken. The KUB showed gaseous distension without free air or evidence of pneumatosis. The following day, the hypertriglyceridemia (Table 1) had resolved, and the NB was started on SMOFlipids. At 25.7 weeks AGA, intermittent hypertriglyceridemia (Table 1) within acceptable limits was seen, and feedings were resumed. The blood urea nitrogen (BUN) (Table 1) level was elevated on routine labs, leading to the PN protein level being maintained at a lower amount. At 27.0 weeks AGA, KUB (Figure 2) showed worsening bowel gas. Therefore, a decompression tube was inserted, with significant bowel gas improvement seen by KUB (Figure 3) at 27.4 weeks AGA. At 28.0 weeks AGA, feeds were restarted and slowly increased to full feeds. Lipids were discontinued at 29.2 weeks AGA.

**Table 1:** Laboratory investigations between 25.5 and 29.2 weeks' AGA AGA: adjusted gestational age

Parameters	Patient Values	Reference Range
Triglycerides	716 mg/dL	0-179 mg/dL
Triglycerides	60 mg/dL	0-179 mg/dL
Triglycerides	226 mg/dL	0-179 mg/dL
Blood Urea Nitrogen	61 mg/dL	5-15 mg/dL

**Figure 4:**
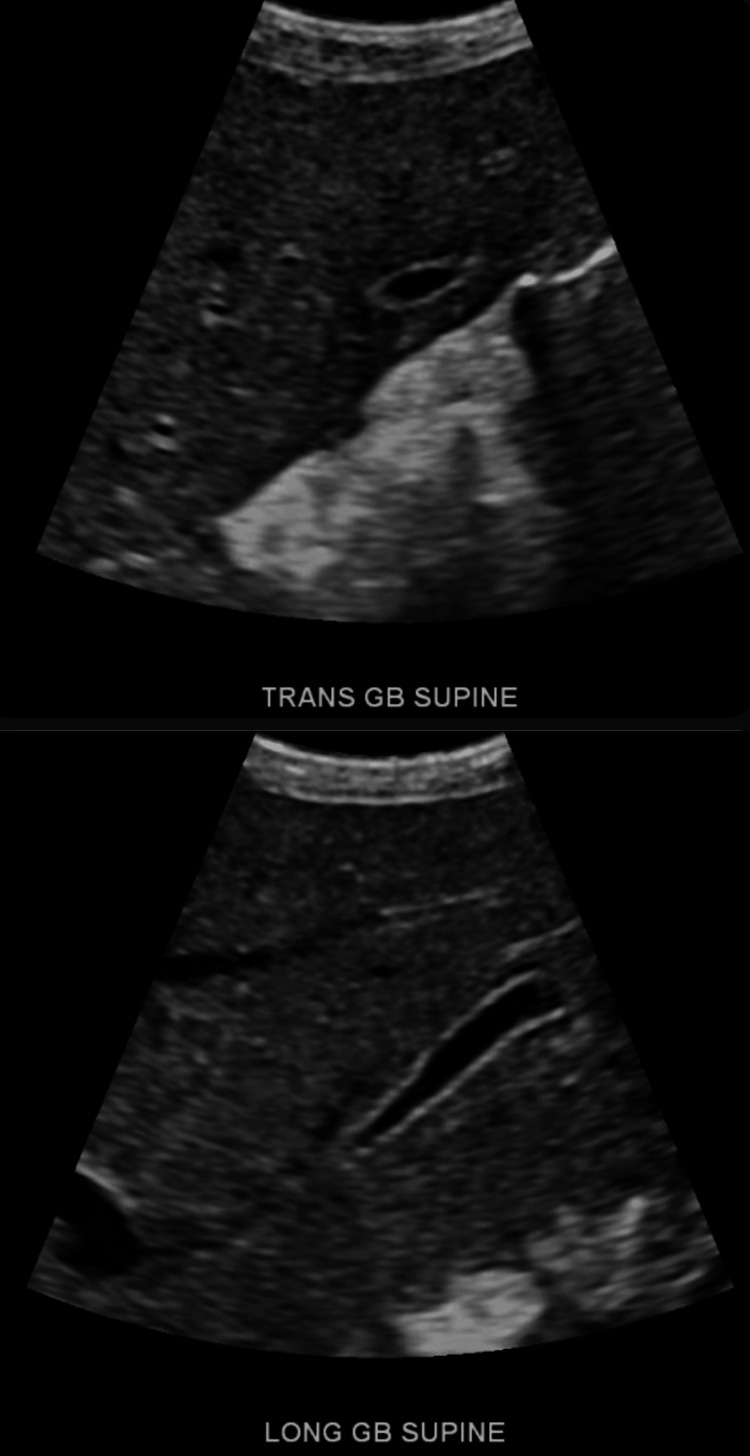
Supine transverse and longitudinal ultrasound views of the gallbladder showing a small, shrunken, and decompressed but not collapsed gallbladder, with no evidence of gallstones, biliary sludge, or biliary obstruction

At 29.5 weeks AGA, the NB continued to have direct hyperbilirubinemia (Table 2), for which she was started on ursodiol, multivitamins, and fat-soluble vitamins the following day. At 30.5 weeks AGA, an abdominal ultrasound (US) (Figure 4) was completed, which showed a partially decompressed gallbladder. This led to a GI consult recommending urinalysis (UA), thyroid-stimulating hormone, and total T4 (Table 2). Additionally, the NB screen (Table 2) came back normal. At 31.1 AGA, the UA (Table 2) showed a positive urinary tract infection (UTI) that was thought to be a possible source for the direct hyperbilirubinemia.

**Table 2:** Laboratory investigations between 29.5 and 31.1 weeks' AGA AGA: adjusted gestational age

Parameters	Patient Values	Reference Range
Direct Bilirubin	6.3 mg/dL	0-0.4 mg/dL
Urinalysis	>100,000 cfu/mL *Escherichia coli*	0 cfu/mL
Thyroid-Stimulating Hormone	1.529 uIU/mL	1.120-6.310 uIU/mL
Total T4	7.1 ug/dL	6.0-12.2 ug/dL
California Newborn Screen	Negative	Negative

The UTI was treated with ampicillin and gentamicin, and repeat TPN labs were ordered for monitoring, and she was started on Pedialyte for dehydration. Between 32.1 and 32.5 weeks AGA, labs showed downtrending direct bilirubin, abnormal FE1, and positive stool reducing substances (Table 3). These abnormal lab results prompted a recommendation from the consulting gastroenterologist to initiate medium-chain triglycerides (MCT) and Neocate formula, as well as to order a cholestasis panel and CF studies (Table 4). At 34.4 weeks AGA, repeat FE1 (Table 3) came back abnormal, and the NB continued to have poor weight gain, so she was started on immobilized lipase. At 34.6 weeks AGA, three CFTR mutations of uncertain clinical relevance were found in the cholestasis panel/CF studies (Table 4), prompting further workup with parental gene analysis the following week.

**Table 3:** Laboratory investigations between 32.1 and 34.6 weeks' AGA AGA: adjusted gestational age

Parameters	Patient Values	Reference Range
Direct Bilirubin	5.7 mg/dL	0-0.4 mg/dL
Pancreatic Elastase 1	121 mcg/g	>200 mcg/g
Stool Reducing Substances	2+ (0.75 g/dL)	Negative
Fecal Elastase 1	72 mcg/g	>200 mcg/g

**Table 4:** Three CFTR gene variants of uncertain clinical significance

Gene, Transcript	Mode of Inheritance, Gene OMIM	DNA Variants, Predicted Effects, Zygosity	ClinVar ID	Highest Allele Frequency in a gnomAD Population	In Silico Missense Predictions	Interpretation
CFTR, NM_000492.3	AD, AR, 602421	c.1327G>T, p.Asp443Tyr, Heterozygous	53229	0.048% European (Non-Finnish)	Damaging	Uncertain
CFTR, NM_000492.3	AD, AR, 602421	c.1727G>C, p.Gly576Ala, Heterozygous	7165	0.78% European (Non-Finnish)	Conflicting	Uncertain
CFTR, NM_000492.3	AD, AR, 602421	c.2002C>T, p.Arg668Cys, Heterozygous	35835	0.94% European (Non-Finnish)	Damaging	Uncertain

The NB continued to have poor weight gain despite the immobilized lipase. It was not until 35.5 weeks AGA that MCT and pancreatic enzyme replacement therapy (PERT) with pancrelipase were started. The NB began to have improved weight gain over the following weeks. At 38.1 weeks AGA, the parental gene analysis confirmed carrier status, as the father had identical mutations. At 38.3 weeks AGA, genetics recommended a rapid exome sequence (Table 5) for other variants and CF “look-alikes.” At 39.6 weeks AGA, the rapid exome sequencing returned negative. Over the past few weeks, three attempts were made to obtain sweat chloride collections, but were unsuccessful. At 40.6 weeks AGA, FE1 had normalized (Table 5), and MCT was titrated down. At 41.4 weeks AGA, PERT was discontinued due to improved weight gain and normalized direct bilirubin (Table 5). A G-tube was inserted at 42.5 weeks AGA, with a postoperative period that was uneventful, and the patient was discharged home with her parents for close follow-up at the high-risk infant clinic.

**Table 5:** Laboratory investigations between 35.5 and 42.5 weeks' AGA AGA: adjusted gestational age

Parameters	Patient Values	Reference Range
Rapid Exome Sequence	Negative	Negative
Pancreatic Elastase 1	743 mcg/g	>200 mcg/g
Direct Bilirubin	0.3 mg/dL	0-0.4 mg/dL

## Discussion

Understanding the pancreas’s development is crucial because abnormalities in embryonic formation, enzyme maturation, or genetic regulation can lead to lifelong pancreatic disease and EPI. Pancreatic development begins between days 26 and 31 of gestation with the formation of dorsal and ventral buds from the foregut endoderm. The ventral bud rotates to fuse with the dorsal bud by the sixth week, producing the mature organ. The ventral bud forms the uncinate process and part of the head, while the dorsal bud forms the body and tail. Ductal formation results in the main pancreatic duct of Wirsung and the accessory duct of Santorini [6].

On a microscopic level, pancreatic tissue develops through the folding and branching of epithelial cells, followed by the differentiation of these cells into acinar, ductal, and endocrine cells. By the ninth week, tubules and clusters of undifferentiated epithelial cells are present, progressing to lobule formation by the 14th week. Acinar cells containing zymogen granules emerge by 12-15 weeks and become numerous by 20 weeks. Pancreatic stellate cells play a fundamental role in early differentiation by secreting extracellular matrix proteins that promote acinar development. Neural networks also form, with intrinsic ganglia and autonomic innervation regulating endocrine and exocrine activity. Parasympathetic vagal input strongly stimulates enzyme secretion, whereas sympathetic input reduces secretion indirectly by limiting blood flow [6].

Exocrine enzyme production begins during the second trimester, but functional maturation continues into infancy. Proteases such as trypsinogen, chymotrypsinogen, and elastase appear by 14-16 weeks of gestation, while carboxypeptidases and lipases are also detectable prenatally. However, pancreatic triglyceride lipase, the primary enzyme for adult fat digestion, does not reach functional levels until after birth. Amylase is unique because it is absent in the fetus and only begins to appear weeks after birth, with adult levels between six months and two years of age. This immaturity explains the “physiologic steatorrhea” observed in neonates, where fat absorption is inefficient. Breast milk compensates through enzymes such as bile salt-stimulated lipase (BSSL) and salivary amylase, while brush-border glucoamylase supports carbohydrate digestion [6].

At the genetic level, pancreatic development is tightly controlled by transcription factors and signaling pathways. Genetic disorders are a significant cause of EPI in children. The primary regulators of acinar and ductal cell lineages responsible for EPI and hypoplasia are GATA4, SOX9, and HNF1B [6]. CF is the most common genetic cause of EPI, resulting from CFTR mutations that lead to thick secretions and duct obstruction, affecting up to 85% of patients [4-6].

EPI leads to the inability to absorb long-chain, calorie-dense fats and fatty acids (FAs) [7]. It is characterized by impaired digestion of macronutrients, particularly fat-soluble vitamins, as a consequence of inadequate delivery of pancreatic enzymes into the duodenum [8]. EPI is statistically defined as postprandial enzyme output <10% of normal [8]. This impaired digestion leads to fat malabsorption, negatively affecting children's growth [7]. EPI is highly correlated with variants of the CFTR gene [8].

Symptoms of EPI are not specific and can include abdominal pain, diarrhea, bloating, and increased appetite [7]. EPI contributes to steatorrhea and greater caloric use than gain [7]. This can lead to malnutrition, deficiencies in trace elements/fat-soluble vitamins, and impaired bone health [7]. Extremely preterm infants are at high risk for GI complications due to developmental immaturity, dysmotility, and the need for PN in the early postnatal period [9,10]. Early advancement of enteral feeds is essential to minimize PN-associated liver disease (PNALD) and support gut maturation [9-11]. Still, feeding intolerance and risk of necrotizing enterocolitis (NEC) often limit early advancement of enteral feeds [9,10]. Human milk is preferred, but preterm formulas with tailored fat compositions are used when human milk is unavailable or insufficient.

A fat balance study can assess pancreatic enzyme function by assessing dietary fat intake and subsequent fat excretion over three days, which will determine the difference in fat absorption [8]. FE1 is a reliable and straightforward lab marker of exocrine pancreatic function from two weeks of age and older [5,8,10,12-15]. Testing for EPI using FE1 should avoid meconium samples and consider the infant’s gestational age and feeding status, as testing on meconium can have a falsely decreased FE1 result [11].

FE1 concentration above 200 μg/g stool is normal, FE1 concentrations below 100 indicate severe EPI, and between 100 and 200 μg/g stool is inconclusive [5,8,10,15]. FE1 concentration may be falsely low in cases of diarrhea due to stool dilution [8]. It is also important to note that FE1 is not affected by PERT. Some patients who are initially pancreatic sufficient later become EPI if they have CF, because CF disease causes progressive pancreatic damage [8]. Repeat testing for EPI using FE1 should consider the infant’s gestational age and feeding status, as FE1 reaches normal levels by day 3 in term infants and by two weeks in very preterm infants [12]. Similarly, earlier gestational age and delayed feeding are associated with a longer time to reach normal FE1 levels [12].

MCTs are an essential component of nutritional support in preterm infants. MCTs are absorbed directly into the portal circulation, bypassing micellar solubilization and bile salt-dependent absorption, making them valuable in fat malabsorption due to pancreatic or hepatic dysfunction [12,13]. In preterm infants, MCT-containing formulas have been shown to reduce stool volume and improve fat and nitrogen absorption [13]. Additionally, when on MCT, less lipase replacement is needed. However, a recent Cochrane review found no significant difference in short-term growth outcomes between high- and low-MCT formulas, and the certainty of evidence to use MCTs remains low [14].

Preterm infants, especially those born extremely preterm, often exhibit transient EPI, with low FE1 levels and associated growth failure [10,15]. The Cystic Fibrosis Foundation recommends initiating PERT in infants with two CFTR mutations associated with EPI, FE1 <200 μg/g, or unequivocal signs of malabsorption while awaiting confirmatory testing [5]. PERT should not be started for neonates with CFTR mutations of unknown significance unless there is objective evidence of EPI or apparent clinical symptoms of malabsorption [5,16]. PERT is essential to maintain adequate nutritional status in EPI neonates. PERT combines lipase, protease, and amylase enzymes to treat EPI due to CF and other GI disorders [8]. When indicated, PERT should be started at 2,000-5,000 lipase units per feeding, not exceeding 2,500 units/kg/feeding or 10,000 units/kg/day, as per Cystic Fibrosis Foundation guidelines [5]. Pancreatic enzymes are administered orally, typically as enteric-coated porcine-derived tablets or microspheres, thereby preventing their inactivation by gastric acid. PERT should be administered with all feeds, including breast milk, elemental, and MCT-containing formulas [7,8,12]. In preterm neonates with growth failure and low FE1, exogenous digestive enzyme replacement (including liquid formulations suitable for gavage) has been associated with improved weight gain [15]. The short-term effectiveness of PERT is well established in clinical practice.

There is a lack of evidence on the optimal timing for starting PERT and the dose of enzyme replacement therapy based on the severity levels of EPI [8]. However, the available evidence does not support routine use of enzyme supplementation in all preterm infants. It should be reserved for those with documented EPI or persistent growth failure despite adequate caloric intake [10,15]. Despite not being approved, oral PERT is commonly used with enteral feeding. Alternatives to porcine-derived PERT are currently under evaluation [8].

Immobilized or recombinant lipase supplementation has been investigated to improve fat absorption in preterm infants, particularly when human milk is pasteurized (inactivating endogenous BSSL) [17,18]. With this goal in mind, an immobilized lipase cartridge (ILC) was developed for extracorporeal digestion of enteral feedings. Recently, studies have demonstrated the safety, tolerability, and efficacy of FA absorption in facilitating fat digestion with continuous enteral feedings. A single-use digestive cartridge containing immobilized lipase (RELiZORB) connects in line with an enteral feeding tube. As enteral feeds pass through the cartridge, the immobilized lipase enzyme hydrolyzes the intact triglyceride fats within the feed into a more absorbable form, with the lipase retained within the cartridge. There was more improvement in growth parameters in the first six months of ILC utilization compared with the latter six months. However, this may be attributed to catch-up growth, which enables better nutrient absorption and a more consistent pattern of pulsatile growth in children. After using an ILC for one year, patients experienced a significant and stable increase in height and weight, along with a consistent improvement in body mass index. Using ILC has demonstrated improvements in eicosapentaenoic acid (EPA) and docosahexaenoic acid (DHA) profiles for premature neonates. Additionally, using an ILC resulted in an increase in hydrolyzed triglycerides in enteral formulas, which led to a decrease in undigested nutrients in the gut lumen [7].

A large randomized trial of recombinant human BSSL in preterm neonates did not show a significant improvement in overall growth velocity. However, a benefit was observed in a subgroup of small-for-gestational-age infants. The safety profile was less favorable, with a higher incidence of adverse events in the treatment group [17,18].

The presence of CFTR mutations of unknown significance in a preterm infant with GI symptoms warrants an evidence-based approach. Diagnosing CF requires clinical manifestations and objective evidence of CFTR dysfunction (e.g., positive sweat chloride or two disease-causing mutations) [16]. Approximately 20% of infants with inconclusive newborn screening results will later meet diagnostic criteria for CF [16]. For infants with CFTR variants of uncertain significance, ongoing clinical follow-up is recommended, including repeat assessment of pancreatic function via FE1, as well as close monitoring for the development of GI or hepatic symptoms [5,16].

Potential adverse effects of MCTs in extremely preterm infants include GI intolerance, such as abdominal distension, diarrhea, or increased gastric residuals [14,18,19]. However, most studies report no significant difference in adverse GI events compared to low-MCT formulas [14,18,19]. There is no clear evidence of an increased risk of NEC with high-MCT formulas; however, the data are limited and of low certainty [14,18,19]. Exclusive use of MCTs can lead to essential FA deficiency if not balanced with long-chain triglycerides; high concentrations may result in incomplete oxidation and elevated dicarboxylic acids, with unclear clinical significance [14,19,20]. MCTs do not appear to increase the risk of cholestasis or hepatic dysfunction in preterm infants, but they do not prevent PN-associated cholestasis [14,18,19].

PERT is generally well tolerated in infants with EPI. Adverse effects include GI symptoms such as abdominal pain, diarrhea, constipation, flatulence, nausea, and skin reactions such as rash, pruritus, and urticaria [5,20]. Rare risks include fibrosing colonopathy, a complication associated with chronic high-dose use (>10,000 lipase units/kg/day), distal intestinal obstruction syndrome (DIOS), hyperuricemia, and allergic reactions to porcine proteins [5,19,20]. The Cystic Fibrosis Foundation recommends dosing not to exceed 2,500 lipase units/kg/feeding or 10,000 units/kg/day to minimize these risks [5].

In infants with CFTR mutations of unknown significance, PERT should only be used if there is objective evidence of EPI or unequivocal malabsorption, as unnecessary enzyme therapy exposes infants to the aforementioned risks without benefit, according to the Cystic Fibrosis Foundation guidelines [5].

Immobilized lipase is not a recognized therapy in this context. All enzyme therapy should be guided by objective evidence of EPI, especially in infants with CFTR mutations of uncertain significance [5].

Stem cell research holds promise for both exocrine and endocrine disorders. Induced pluripotent stem cells (iPSCs) may one day be differentiated into pancreatic progenitor cells for use in patients with diabetes or EPI [6]. Advances in whole-exome sequencing also reveal new variants associated with pancreatic dysfunction, which may inform personalized care. Key unanswered questions remain, including the timing of autonomic innervation development, the triggers for the maturation of enzyme secretion, and the role of pancreatic stellate cells in guiding cell interactions.

## Conclusions

This patient presented with a mixed presentation; their exocrine pancreatic enzymes did not improve over time on their own, which would indicate immaturity, and required PERT. However, they were subsequently able to produce pancreatic exocrine enzymes, did not require further supplementation, and the pancreatic enzymes had stabilized on future laboratory tests. EPI in extremely preterm infants is uncommon and diagnostically challenging, requiring distinction from physiologic enzyme immaturity. In this case, persistent malabsorption and growth faltering, confirmed by serial FE1 testing, warranted initiation of MCTs, PERT, and ILCs, which resulted in clinical improvement through growth and development. The discovery of CFTR variants of uncertain significance underscores the importance of ongoing genetic and functional research into the effects of CFTR variations. Early recognition of EPI and targeted therapy is crucial for optimizing growth and development outcomes in neonatal patients.

Adjunctive therapies such as MCTs, PERT, and ILCs offer potential benefits in supporting growth and nutrient absorption in patients with EPI. Still, their use must be guided by objective evidence of EPI. Each therapy has its limitations and potential adverse effects, including GI intolerance, essential FA deficiency, or rare complications from high-dose enzyme therapy. Therefore, careful dosing, ongoing monitoring, and selective treatment selection are crucial, particularly in infants with CFTR variants of uncertain significance.
